# Alpha/beta-gamma decoupling in methylphenidate medicated ADHD patients

**DOI:** 10.3389/fnins.2023.1267901

**Published:** 2023-09-29

**Authors:** Nowell Zammit, Richard Muscat

**Affiliations:** ^1^Centre for Molecular Medicine and Biobanking, University of Malta, Msida, Malta; ^2^Department of Physiology and Biochemistry, University of Malta, Msida, Malta

**Keywords:** methylphenidate, EEG, neural oscillations, ADHD, coupling, working memory

## Abstract

There is much interest to understand how different neural rhythms function, interact and are regulated. Here, we focus on WM delay gamma to investigate its coupling with alpha/beta rhythms and its neuromodulation by methylphenidate. We address this through the use of human EEG conducted in healthy and ADHD subjects which revealed ADHD-specific electrophysiological deficits and MPH-induced normalization of gamma amplitude and its coupling with alpha/beta rhythms. Decreased alpha/beta-gamma coupling is known to facilitate memory representations via disinhibition of gamma ensembles coding the maintained stimuli. Here, we present EEG evidence which suggests that these dynamics are sensitive to catecholaminergic neuromodulation. MPH decreased alpha/beta-gamma coupling and this was related to the increase in delay-relevant gamma activity evoked by the same drug. These results add further to the neuromodulatory findings that reflect an electrophysiological dimension to the well-known link between WM delay and catecholaminergic transmission.

## 1. Introduction

It is said that “life can only be understood backwards, but must be lived forwards” (Soren Kierkegaard). Such faculties revolve around the ability to maintain neural representation of events that defeat time and persist long after these dissipate from the sensorium. Without this elementary capability, much of our sophisticated cognition fades away, and with it, much of the high-order cortical circuitry reserved for manipulating and processing dissipated sensory information. The concept of sustained activity was ignited following the seminal studies by Fuster and Alexander (1971) and Kubota and Niki (1971), coining the term “delay activity” to prefrontal working memory (WM) activity that over-lasts sensory stimulation. Following these findings, studies have recorded such activities from various other cortical (e.g., parietal, temporal and sensory cortices: Miyashita and Chang, 1988; Ranganath and Blumenfeld, 2005; Meltzer et al., 2008) and sub-cortical regions (e.g., thalamic and basal ganglia nuclei: Alexander, 1987; Watanabe and Funahashi, 2012) hinting at the possibility that such delay-dynamics might be shared across regions (Sreenivasan and D’Esposito, 2019). Recent developments in the characterization of laminar circuits have put forth the need to incorporate existing WM delay models within the detailed architectures that comprise interlaminar connectivity. Laminar recordings suggest a functional division between superficial and deep cortical layers, as in fact, superficial layers feedforward bottom-up signals, while deep layers feedback top-down signals (Bastos et al., 2020). This laminar segregation is also apparent at the electrophysiological level, as both superficial and deep layers endorse their own frequency bandwidths to transmit bottom-up and top-down information, respectively (Bastos et al., 2015, 2018).

Top-down regulation of sensory activity is most likely implemented by activating a default inhibitory mechanism that regulates which bottom-up signals are feedforwarded up the cortical hierarchy (Pinotsis and Miller, 2020). In this framework, WM maintenance reflects a state of disinhibition that allows both the representation and persistence of superficially encoded sensory information. In a series of experiments conducted on primates (Bastos et al., 2018), the delay period activity in a delayed-matching to sample task (DMTS) was found to be dominated by superficial activity that was largely composed of gamma band perturbations. Cross frequency coupling revealed that this delay-evoked gamma was significantly decoupled from the deeper layer alpha/beta activity. Decoupling presumably released the superficial layer from the inhibitory influence mediated by the slower rhythms, as indeed, delay-gamma and alpha/beta activities were found to be negatively correlated. Although it remains to be known whether the variations exhibited in decoupling strength are associated directly with the amplitude changes in the decoupled gamma bands, however, cortical circuits might appear to exploit interlaminar coupling as a mechanism to select which representation should be maintained or not.

There have been various attempts to characterize the neurotransmission characteristics within the underlying circuits that give rise to WM delay activity and among others, the catecholaminergic neurotransmitters, dopamine and noradrenaline, have received much attention. The role played by dopaminergic neurons within these circuits in WM delay has been dominated by investigations that have targeted D1 receptor signaling and its corresponding inverted U-dose response relationship with WM performance. Studies (e.g., Vijayraghavan et al., 2007; Wang et al., 2019) have shown that the inverted U-response is substantiated by a mechanism that involves D1-induced excitatory/inhibitory perturbations that optimize stimulus tuning in an inverted U-dose response manner. Thus, beyond mere stimulus maintenance, D1 signaling appears to regulate which neuronal ensembles participate in the delay activity by biasing selection toward the relevant stimuli (Jacob et al., 2016). This regulatory role is not unique to dopamine D1 signaling as this is also evoked by noradrenergic transmission, particularly, the adrenergic α2-receptor which is known to dampen distractibility during WM delay (Arnsten and Contant, 1992; Arnsten, 2006).

The above would appear to suggest that there may be some overlap with the selectivity invoked by the laminar gamma-alpha/beta model and therefore raises the question of whether the catecholaminergic role in the selection of delay representations might materialize in the form of some sort of regulation in the gamma-alpha/beta coupling. Both gamma and alpha/beta activities are known to be modulated by catecholaminergic transmission (Neves et al., 2018; Zaldivar et al., 2018), however, whether such modulation is coordinated across the two bands in the service of memory delay, remains to be seen.

Methylphenidate (MPH) is a psychostimulant drug, which has been shown to enhance various cognitive functions, including that of WM (Mehta et al., 2004; Linssen et al., 2014). MPH blocks catecholamine transporters, evoking higher neurotransmitter concentrations of both dopamine and noradrenaline (Berridge and Stalnaker, 2002). Although a complete characterization of which receptors are activated by MPH remains elusive, however, it also appears that MPH effects can be reversed by specific dopaminergic and noradrenergic receptor blockers. The dopamine D1 and noradrenaline α2 receptors have been revealed to mediate much of MPH’s effects, as in fact, oral administration of therapeutically relevant doses of MPH evoked WM improvements that were entirely reversed by dopamine D1 and adrenergic α2 receptors antagonists (Arnsten and Dudley, 2005).

MPH is the leading treatment for Attention Deficit Hyperactivity Disorder (ADHD), a neurobehavioral disorder characterized by inattention and hyperactivity (American Psychiatric Association, 2013). Much of MPH’s propensity to reverse ADHD symptomology (Prince, 2008) revolves around findings that support ADHD as a catecholaminergic disorder (Vaidya and Stollstorff, 2008). This convergence appears to be supported by studies which relate ADHD and MPH to a common cluster of cognitive functions, including attention (Cox et al., 2004), response inhibition (Vaidya et al., 1998), and WM (Strand et al., 2012). The study of clinical ADHD patients undergoing MPH treatment opens up a window of opportunity to assess whether and how catecholamines might modulate human WM delay activity. In particular, this might shed further light as to whether catecholaminergic transmission during human WM delay might play a role in the modulation of gamma-alpha/beta coupling.

Laminar fMRI reveals that much of the WM delay activity in humans originates from the superficial cortical layers (Finn et al., 2019). If WM delay mechanisms in humans are homologous to those in primates, the stimulation of WM delay is likely to invoke superficial cortical activity which presumably “rings” back with a corresponding perturbation in the gamma range. Human Electroencephalogram (EEG) gamma is sampled from superficial cortical layers (Scheeringa et al., 2016), suggesting a good probability that WM delay gamma might be more likely than not detectable during human EEG recordings. Here, we sought to investigate the latter by presenting typically developing (TD) and MPH-medicated ADHD adolescent subjects with a visual DMTS task. Gamma oscillations are associated with multiple cognitive states (Basar, 2013) and teasing these apart requires a control condition that replicates the DMTS task without requiring subjects to memorize the stimulus items, which we here refer to as the WM-CTR task. The delay period in the DMTS task evoked a significant gamma enhancement in frontal–parietal electrodes that was not evident in the WM-CTR task, thus implying WM-specificity. The stronger delay gamma activity elicited by MPH could in various instances be explained by the association of a weaker gamma-alpha/beta coupling induced by the same drug. The modulation of WM-delay gamma and its decoupling with the alpha/beta rhythms represents yet another electrophysiological mechanism that pieces together the various of several ways through which catecholamines might substantiate WM.

## 2. Methods

### 2.1. Participants

In the current study, we analyse unpublished EEG data, obtained from a sample of 30 male subjects (15 with ADHD) aged between 12 and 14 years of age (TD: mean age = 13.13 years, S.E.M = 0.227) (ADHD: mean age = 13.39 years, S.E.M = 0.189), recruited as part of our previous human EEG-WM study (Zammit and Muscat, 2019). Briefly, subjects were recruited into the TD group if they were in general good health, had no history of psychiatric or neurological illness and scored <65 t-score on the global ADHD index of the Conners parent and teacher rating scales (Conners, 1997) (Table 1). Participants were recruited into the ADHD group if they met the DSM-V criteria for ADHD conducted by a state-certified clinician, obtained a t-score of >65 on the global index of the Conners parent and teacher rating scales, were not diagnosed with any other comorbid disorder and/or prescribed psychoactive medications, other than oral MPH (Ritalin—immediate release) medication for a period of at least 1 year. ADHD subjects performed the DMTS task during simultaneous EEG recordings on two separate occasions. On one of the occasions, referred to as “ON-medication” condition, EEG recordings from subjects were conducted in the morning, 2 h following ingestion of a 10 mg Ritalin tablet, during when the MPH plasma concentration reaches its peak (Markowitz et al., 2003) while on the other occasion, referred as “OFF-medication” condition, EEG recordings were again conducted in the morning, this time, 12 h following their last 10 mg Ritalin dose, when MPH plasma concentrations return back to baseline (Markowitz et al., 2003). Each of the medicated subjects performed the two medication conditions in a counterbalanced order in that eight of the subjects performed the “ON-medication” condition on the first occasion and the other seven, the “OFF-medication” condition, and on the second occasion this was reversed.

**Table 1:** Sample demographics and symptoms rated by Conners parent and teacher rating scales.

Subject type	*N*	AGE	*T*_OPP_	*T*_ATT_	*T*_HYP_	*T*_GLO_	*P*_OPP_	*P*_ATT_	*P*_HYP_	*P*_GLO_
TD	15	13.13	55.7	51.1	55.7	51.9	56.8	53.9	56.5	50.1
ADHD	15	13.39	76.1	69.2	80.0	75.0	75.4	69.7	82.7	73.7

*T*_OPP_: Conners teacher oppositional index; *T*_ATT_: Conners teacher attentional index; *T*_HYP_: Conners teacher hyperactivity index; *T*_GLO_: Conners teacher global index; *P*_OPP_: Conners parent oppositional index; *P*_ATT_: Conners parent attentional index; *P*_HYP_: Conners parent hyperactivity index; *P*_GLO_: Conners parent global index.

### 2.2. Tasks and procedures

**Figure 1:**
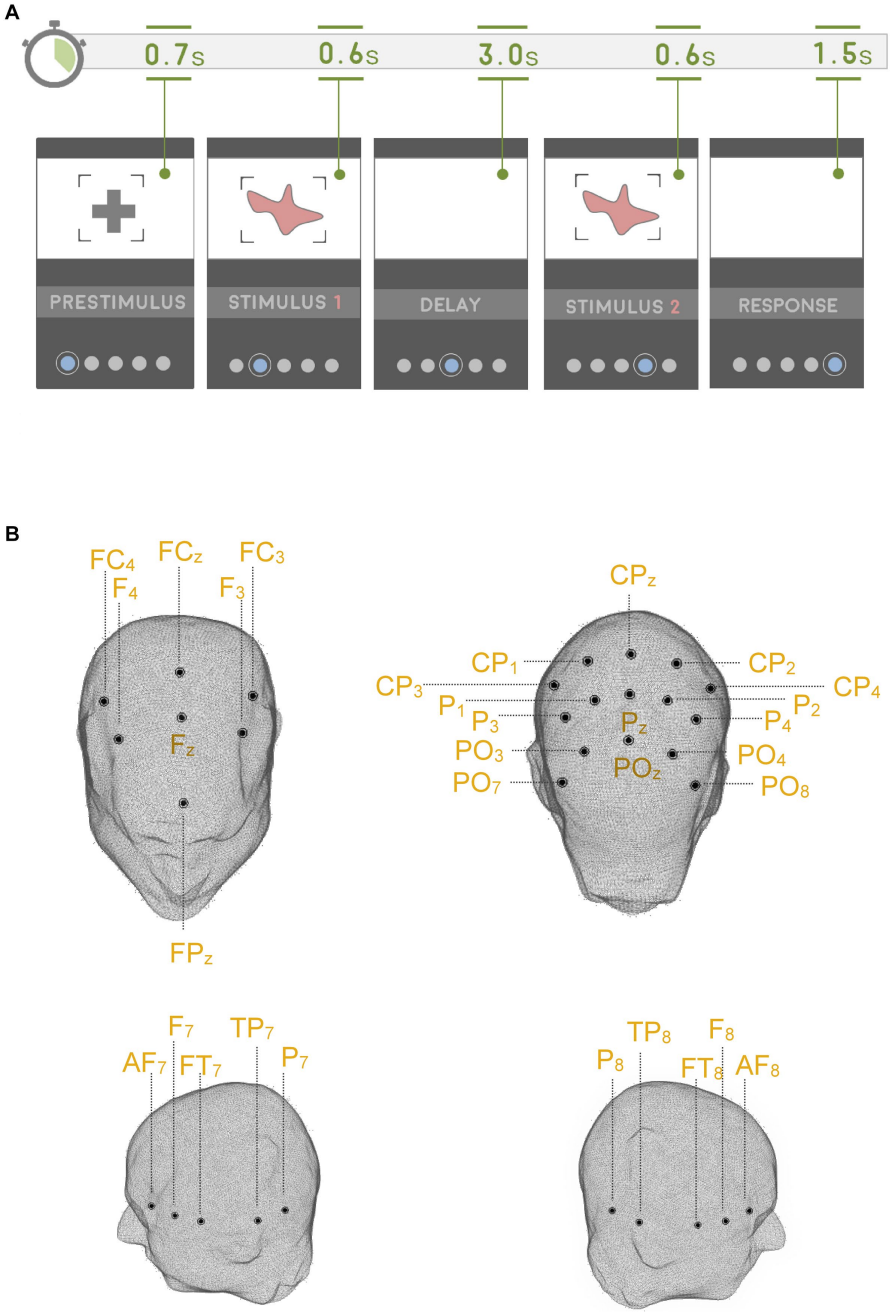
Tasks and electrode configuration. **(A)** DMTS task. The WM-CTR was exactly identical to the DMTS task, except that it lasted up until the delay offset. **(B)** Electrode configuration used in the study.

Subjects performed on a visual DMTS task during simultaneous EEG recordings (Figure 1A). The visual stimuli consisted of abstract black shapes displayed on a white background. Briefly, each trial in the DMTS task began with the delivery of a central fixation cross that was presented for 700 ms. During the encoding phase, a visual stimulus was presented for 600 ms. Next, the visual stimulus period was followed by a 3,000 ms delay period. In the delay period, subjects were presented with a blank screen and were instructed to retain the previous visual stimulus in memory. Following the delay period, a second visual stimulus was delivered for 600 ms, which could either “match” or “un-match” the first visual stimulus. During the response period, subjects had to press a button each time that the first and second visual stimuli matched each other. The response period was set to 1,500 ms.

Each subject performed 250 trials of the DMTS task that were organized into blocks (approximately 8 min per block). As described above, ADHD subjects performed the DMTS task on two occasions (ON & OFF medication conditions). Epochs from incorrect DMTS task trials were not analyzed in this study. TD subjects also performed on the WM-CTR task, in a separate session, which acted as a control task where no retention of the visual stimuli was required. In the WM-CTR task, each trial began with the delivery of a central fixation cross that was presented for 700 ms. Next, a visual stimulus was presented for 600 ms. The visual stimulus period was followed by a 3,000 ms delay period. Subjects performed 250 trials of the WM-CTR task that were organized into blocks (approximately 8 min per block). Although subjects performing the WM-CTR task were assured that they were not being tested on the task, yet they were instructed to pay attention to each and every visual stimulus delivered during the WM-CTR task. In this study, the delay intervals in the WM-CTR task and the DMTS task were the main events of interest. All procedures were approved by the University of Malta Research Ethics Committee and were conducted in accordance with the Declaration of Helsinki.

### 2.3. EEG data acquisition

EEG was recorded using active 32 Ag-AgCl electrodes (Guger technologies—g.tec) distributed across the frontal and parietal scalp regions according to the 10/20 system (please see Figure 1B). EEG signals were acquired using g.GAMMAsys hardware and g.Recorder software (Guger technologies). EEG signals were ear-lobe referenced, sampled at 256 Hz rate and filtered between 1 and 100 Hz (notch filtered at 50 Hz). We utilized g.TRIGbox (Guger technologies) to synchronize the events of the cognitive tasks with the EEG recording, thus “marking” the EEG signal each time that the cognitive events of interest were concurrently displayed on the presentation screen. The g.TRIGbox was connected to an optical sensor which detected a flashing black square that was programmed to dis/appear during the offset and onset of all the cognitive events of interest.

### 2.4. EEG data analysis

Offline EEG data processing was performed using both EEGLAB (Delorme and Makeig, 2004) and Fieldtrip functions (Oostenveld et al., 2011), in addition to custom MATLAB (MathWorks) scripts. Artifact-contaminated signals in the continuous data were initially detected and rejected using both manual inspection and automated EEGLAB plugins; Clean_rawdata (Delorme and Makeig, 2004). Clean_rawdata identified artifact EEG channels if they were flat for more than 5 s, poorly correlated (*r* < 0.8) with surrounding channels and noisy (±5 SD). The data was later segmented into 6,500 ms trial epochs that stretched from −2,000 ms to 4,500 ms of the delay period onset in the DMTS and WM-CTR tasks. A 400 ms interval in the prestimulus period of the DMTS and WM-CTR tasks (from 200 ms to 600 ms of the prestimulus interval, please see Figure 1A) acted as the baseline interval for each trial.

Artifact-contaminated epochs were rejected using both manual visual inspection and EEGLAB in-built functions for improbable data distributions and abnormal peaks in the data (threshold set at ±5 SD). EEG signals were decomposed into independent source components using Independent Component Analysis (ICA) (extended infomax) (Lee et al., 1999) to isolate stereotypic artifacts present in the epoched EEG data. ICA is a linear decomposition method that ‘un-mixes’ the time course of both brain and non-brain source activations that have been mixed by volume conduction and recorded at the scalp EEG electrode level.

Each of the independent components (ICs) was manually inspected for artifact contamination by assessing its spectra, scalp topography and activity across time and trials. Additionally, two EEGLAB plugins were used to distinguish between brain-related ICs and artifact ICs: ADJUST (Mognon et al., 2011) and MARA (Winkler et al., 2011). While ADJUST is mostly concentrated on detecting ocular artifacts, MARA is capable of identifying muscular artifacts as well. The identified artifact ICs were subtracted from the EEG signals.

Next, we computed the time frequency power profiles generated in the epoched data. Single trial frequency power profiles between 1 and 100 Hz, stretching −1,300 ms to 4,000 ms relative to the delay period onset, were estimated by using a Hanning taper method with a fixed 500 ms sliding window length (1 Hz frequency resolution), centered every 50 ms, as implemented in the Fieldtrip toolbox (using the “mtmconvol” function). These estimates were later decibel normalized (dB power = 10*log10 [power/baseline]), using a baseline period, as described above. Three-dimensional topographical scalp plots were computed to visualize the averaged and normalized raw power evoked across the gamma (30–100 Hz) frequency band during the 3,000 ms delay time period. Two-dimensional topographical maps displaying the normalized gamma power across channels were extracted, averaged across subjects and later warped against a template head mesh scalp model, created using fieldtrip functions and aligned with the 3D electrode scalp coordinates.

The relationship between the alpha/beta phase and gamma amplitude was quantified using the PAC, using the modulation index (MI) as described and implemented in a series of Matlab functions made available by Tort et al. (2010). Briefly, the instantaneous phase and amplitude series of slow and fast band-pass filtered signals were extracted using the Hilbert transform and aligned. The phases of the slow-frequencies were organized into 18 bins of 20° steps and the amplitudes of the faster-frequencies were averaged within each of the phase bins. PAC emerges when the amplitudes across the phase bins deviate from a hypothesized uniform distribution, which is quantified using the Kullback–Leibler distance (MI) and normalized such that values range between 0 and 1. MI indices were computed for phases of frequencies that stretched between 5 and 30 Hz (1 Hz steps) and amplitudes of frequencies between 31 and 100 Hz (2 Hz step) across all trials and electrodes. Statistical PAC differences were assessed using the non-parametric, cluster-based permutation method (Maris and Oostenveld, 2007) (please see below). Correlations between alpha/beta-gamma PAC and gamma amplitude during the delay period were computed by indexing those specific gamma frequencies (30–100 Hz) that showed significant coupling differences (across conditions) with either alpha (7–14 Hz) or beta (15–20 Hz) phase. The amplitude of the indexed gamma frequencies was averaged across subject and channel, and correlated with the corresponding MI index obtained for the same gamma frequencies in relation with the alpha or beta phase.

Statistical analyses were performed on Matlab (Mathworks). Behavioral data obtained from the subjects’ performance on the DMTS task were statistically analyzed by computing paired or unpaired *t*-tests, using the Matlab functions: “ttest” and “ttest2.” Pearson correlations between gamma power and DMTS performance, as well between alpha/beta-gamma PAC and gamma amplitude, were performed using the Matlab function “corr.” The statistical differences in the time-frequency and PAC profiles were assessed using the non-parametric, cluster-based permutation method (Maris and Oostenveld, 2007), which adjusts for multiple comparisons (implemented in Fieldtrip toolbox). In this method, statistical comparisons were analyzed by calculating the t-statistic, which was computed separately for each baseline-normalized frequency bin and time point. Statistical analysis was conducted for frequencies within the gamma band range: 30–100 Hz across the 0 ms to 3,000 ms temporal period following delay onset. A threshold was applied to the data, eliminating those with *p*-values above the 0.05 alpha level. Data samples that survived this threshold were then clustered. Clustering was performed separately for positive and negative *t*-values. Clusters were formed by two or more neighboring channels in space, based on the triangulation method. Cluster-based statistics were computed by summing up the *t*-values across each of the various clusters of data samples that were connected in terms of temporal and spectral adjacency (cluster statistic). The Monte Carlo method was used to generate the permutation distribution of the largest cluster statistic. Here, data samples pertaining to the condition or groups under comparison were randomly shuffled 2,000 times and after each randomization, the cluster with the largest sum of *t*-values entered the distribution. Lastly, the two-tailed *p*-values of the actual clusters were obtained by comparing their statistic against the permutation distribution of the largest cluster statistic. A two-sided *p*-value less than 0.025 was considered statistically significant. Cohen’s U1 index was computed, using the MES toolbox in MATLAB, developed for neuroscientific datasets (Hentschke and Stuttgen, 2011), to quantify the effect size for the cluster comparisons.

## 3. Results

### 3.1. Behavioral results

In the DMTS task, subjects were required to determine whether two visual stimuli, separated by a blank delay period, matched each other or not. Among others, correct responses were contingent upon the subjects’ ability to retain visual sensory information during the delay period where the remembered stimulus is no longer available. On average, TD and ADHD subjects in the ‘OFF’ medication condition obtained 83% ± 2.26% (S.E.M) and 65% ± 4.43% (S.E.M) correct responses, respectively. These scores were found to be significantly different (*p* = 0.002), with TD scoring higher on the task. When the same analysis was conducted to check whether MPH modulated the percentage of DMTS correct score within the ADHD patients, findings revealed that MPH triggered a statistically significant increase in the mean DMTS percentage score (76% ± 3.96% (S.E.M), *p* = 0.001), to the extent that this score was not statistically different from that obtained from TD subjects (*p* = 0.148). These results support the conclusion that MPH improves the DMTS performance deficit seen in the non-medicated ADHD cohort to the same levels as those obtained by TD subjects.

### 3.2. EEG results

#### 3.2.1. Delay-specific gamma enhancement

**Figure 2:**
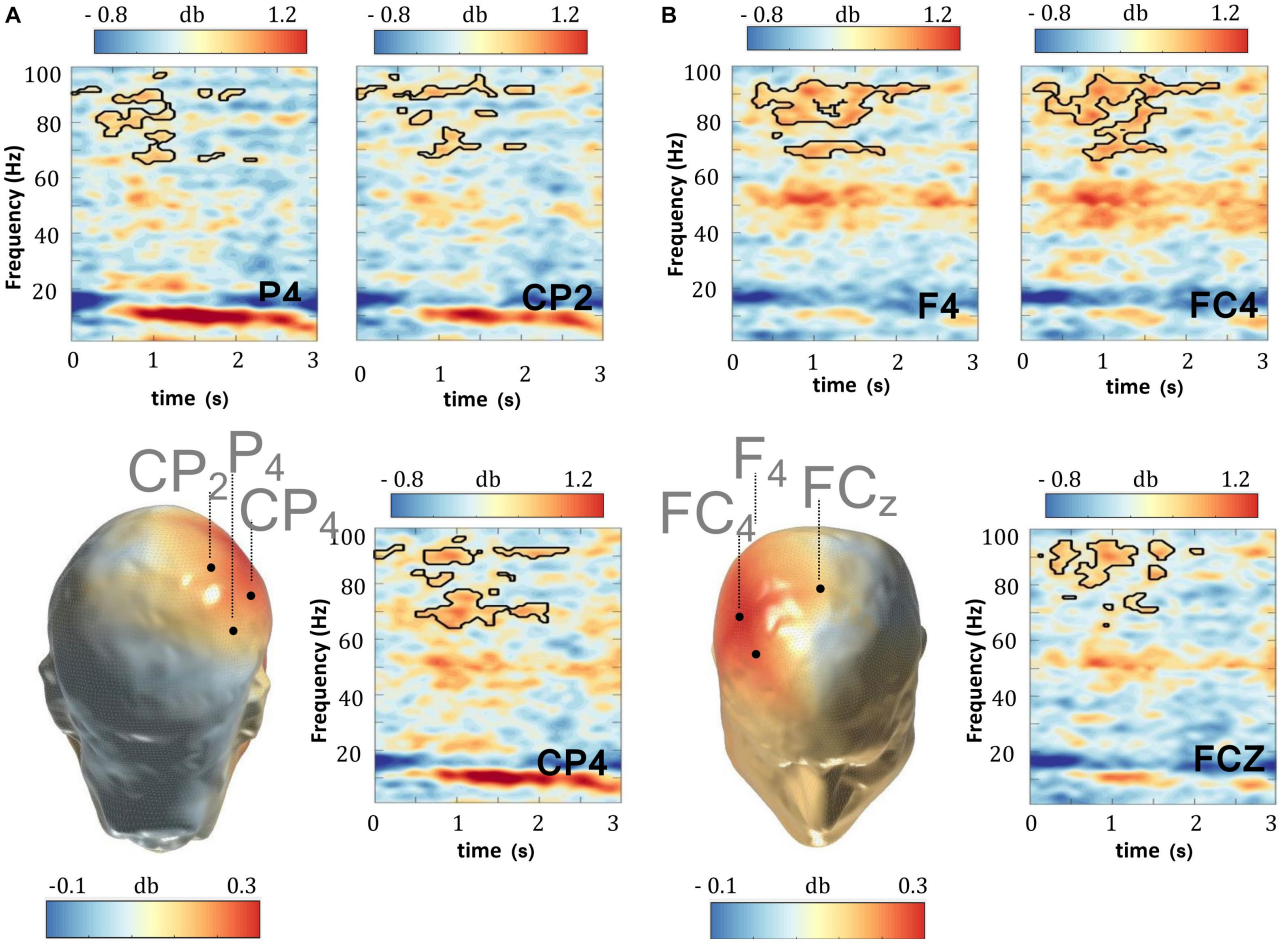
Gamma oscillations and WM delay. In TD subjects, the delay period (0–3,000 ms) of the DMTS task evoked stronger gamma power relative to the delay period (0–3,000 ms) in the WM-CTR task. This effect was pronounced in the **(A)** parietal and **(B)** frontal scalp regions. Scalp maps reflect the averaged gamma power (30–100 Hz) during the 0–3,000 ms window following delay period onset. The outline in the spectrograms demarcates gamma frequencies where statistically significant (two-sided test*, p* < 0.025) enhancements were recorded.

In the first series of analysis, we attempted to probe the oscillatory activity during WM retention in the delay period of the DMTS task. This analysis was focused on those instances when TD subjects were actively engaged in WM retention by contrasting the delay period of the DMTS task with that of the WM-CTR task, which featured the same stimulus events without requiring subjects to hold the stimulus items in memory. The resultant findings replicate those of previous studies in which a significant gamma enhancement (cluster statistic = 14,783, corrected *p* = 0.018, SD = 0.003, Cohen’s U1 = 0.5) across all of the electrodes sampled was found (Figure 2, Supplementary Figure S1). This gamma enhancement was specific to the faster gamma frequency range. No other significant enhancements/decrements in the various other bands were recorded. Since the scalp maps in Figure 2 appear to suggest a lateralized gamma band enhancement, we asked whether such differences might be lateralized to one side of the scalp more than the other. As such, we compared electrodes located on one side of the scalp with their symmetric pairs on the other side (left-side scalp electrodes: *n* = 13, [PO7, PO3, P7, P3, P1, TP7, CP3, CP1, FT7, FC3, F7, F3, AF7]; right-side scalp electrodes: *n* = 13, [PO8, PO4, P8, P4, P2, TP8, CP4, CP2, FT8, FC4, F8, F4, AF8]). As indicated in Table 2, the left and right scalp electrode regions displayed statistically similar gamma power levels.

**Table 2:** Laterality effects for the gamma band (30–100 Hz) between the DMTS and WM-CTR task.

*p*-value		Cluster statistic		SD		Cohen’s U1	
Cl_Pos_	Cl _Neg_	Cl_Pos_	Cl_Neg_	Cl_Pos_	Cl_Neg_	Cl_Pos_	Cl_Neg_
0.03	0.94	1117.4	−161.4	0.004	0.005	0.07	0.07

Left-sided electrodes (*n* = 13): PO7, PO3, P7, P3, P1, TP7, CP3, CP1, FT7, FC3, F7, F3, AF7; Right-sided electrodes (*n* = 13): PO8, PO4, P8, P4, P2, TP8, CP4, CP2, FT8, FC4, F8, F4, AF8. Cl_Pos_: Largest positive cluster; Cl_Neg_: Largest negative cluster. The analysis shows that the comparison was not statistically significant (two-sided test, *p* > 0.025).

#### 3.2.2. Delay-specific gamma deficits in ADHD patients

**Figure 3:**
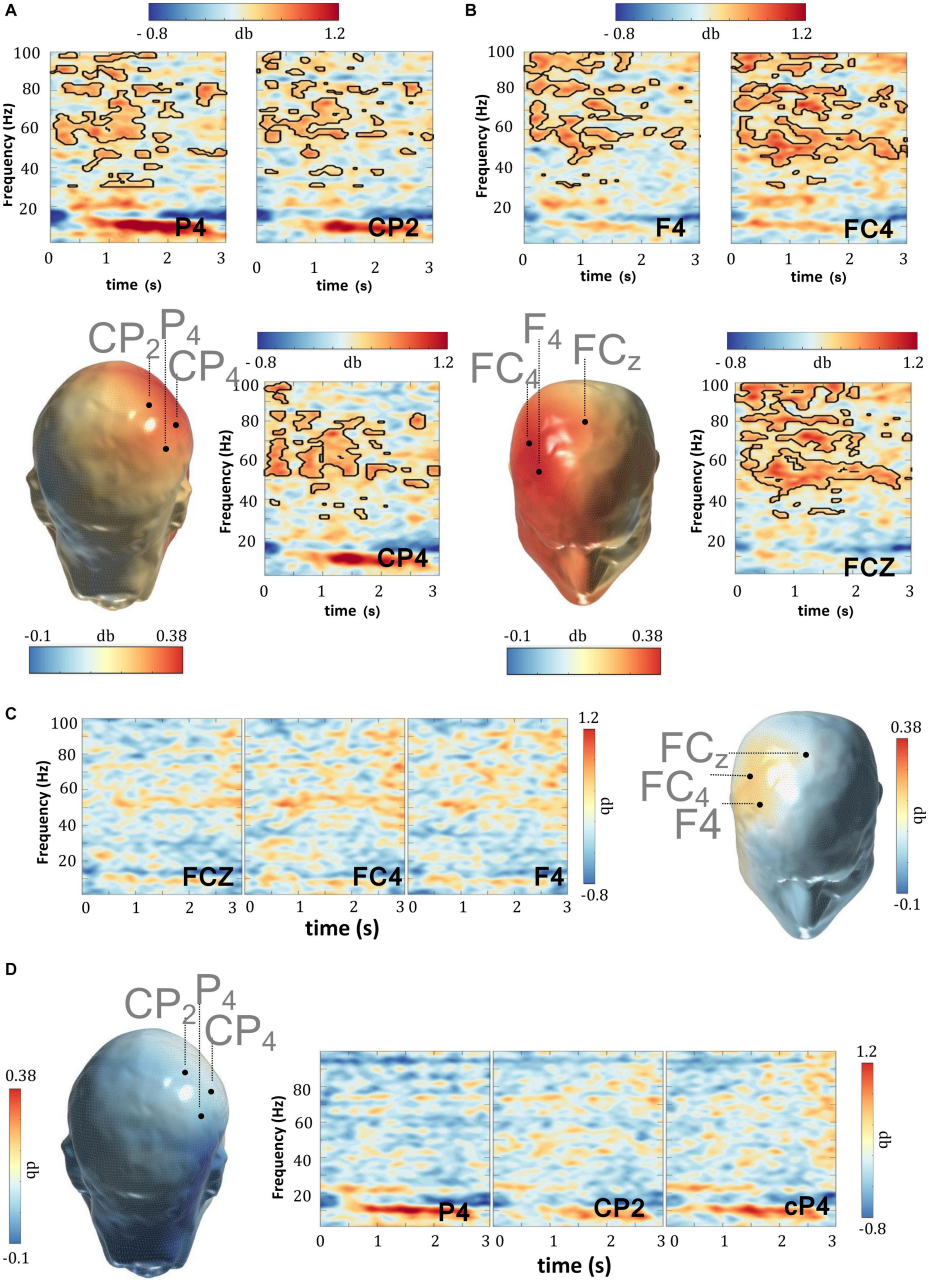
WM delay gamma deficits in ADHD. The delay period (0–3,000 ms) of the DMTS task recorded in TD subjects evoked stronger gamma power relative to the delay period (0–3,000 ms) recorded in ADHD subjects when not under MPH medication. This effect is evident in **(A)** parietal and **(B)** frontal scalp regions. **(C,D)** There were no frequency activity differences during the delay period (0–3,000 ms) of the DMTS task between TD and ADHD subjects when under MPH medication. MPH normalized delay gamma activities in the ADHD subjects. Scalp maps reflect the averaged gamma power (30–100 Hz) during the 0–3,000 ms window following delay period onset. The outline in the spectrograms demarcates gamma frequencies where statistically significant (two-sided test*, p* < 0.025) enhancements were recorded.

The significant enhancements above imply that gamma oscillating cortical circuits might be particularly sensitive to the modulatory effects imparted by WM retention. This in turn, raises the possibility that the weaker WM performance in the ADHD patients herein might be partly explained by co-occurring gamma amplitude deficits evoked during WM-delay. The comparison between TD and ADHD subjects in the “OFF-medication” condition, portrayed in Figures 3A,B and Supplementary Figure S2, indeed revealed that the amplitude of gamma activity elicited during WM retention in the DMTS task was significantly weaker in ADHD patients, relative to TD (cluster statistic = −40,882, corrected *p* = 0.004, SD = 0.001, Cohen’s U1 = 0.6). To investigate whether the difference in the gamma band might constitute a laterality effect, we also compared the differential gamma activity (between the TD and ADHD subjects in the “OFF-medication” condition), across the left (*n* = 13) and right (*n* = 13) electrode regions. When the right and left electrode groups were compared, cluster based permutation statistics yielded no significant differences, implying no gamma differences between the left and right scalp electrodes (Table 3). This suggests that the gamma deficits herein did not distinguish one side of the scalp more than the other.

**Table 3:** Laterality effects for the gamma band (30–100 Hz) between the TD and ADHD subjects in the “OFF-medication condition.”

*p*-value		Cluster statistic		SD		Cohen’s U1	
Cl_Pos_	Cl_Neg_	Cl_Pos_	Cl_Neg_	Cl_Pos_	Cl_Neg_	Cl_Pos_	Cl_Neg_
0.93	0.99	69.1	−22.96	0.005	0.002	0.03	0.03

Left-sided electrodes (*n* = 13): PO7, PO3, P7, P3, P1, TP7, CP3, CP1, FT7, FC3, F7, F3, AF7; Right-sided electrodes (*n* = 13): PO8, PO4, P8, P4, P2, TP8, CP4, CP2, FT8, FC4, F8, F4, AF8. Cl_Pos_: Largest positive cluster; Cl_Neg_: Largest negative cluster. The analysis shows that the comparison was not statistically significant (two-sided test, *p* > 0.025).

#### 3.2.3. Catecholaminergic sources of gamma deficits

**Figure 4:**
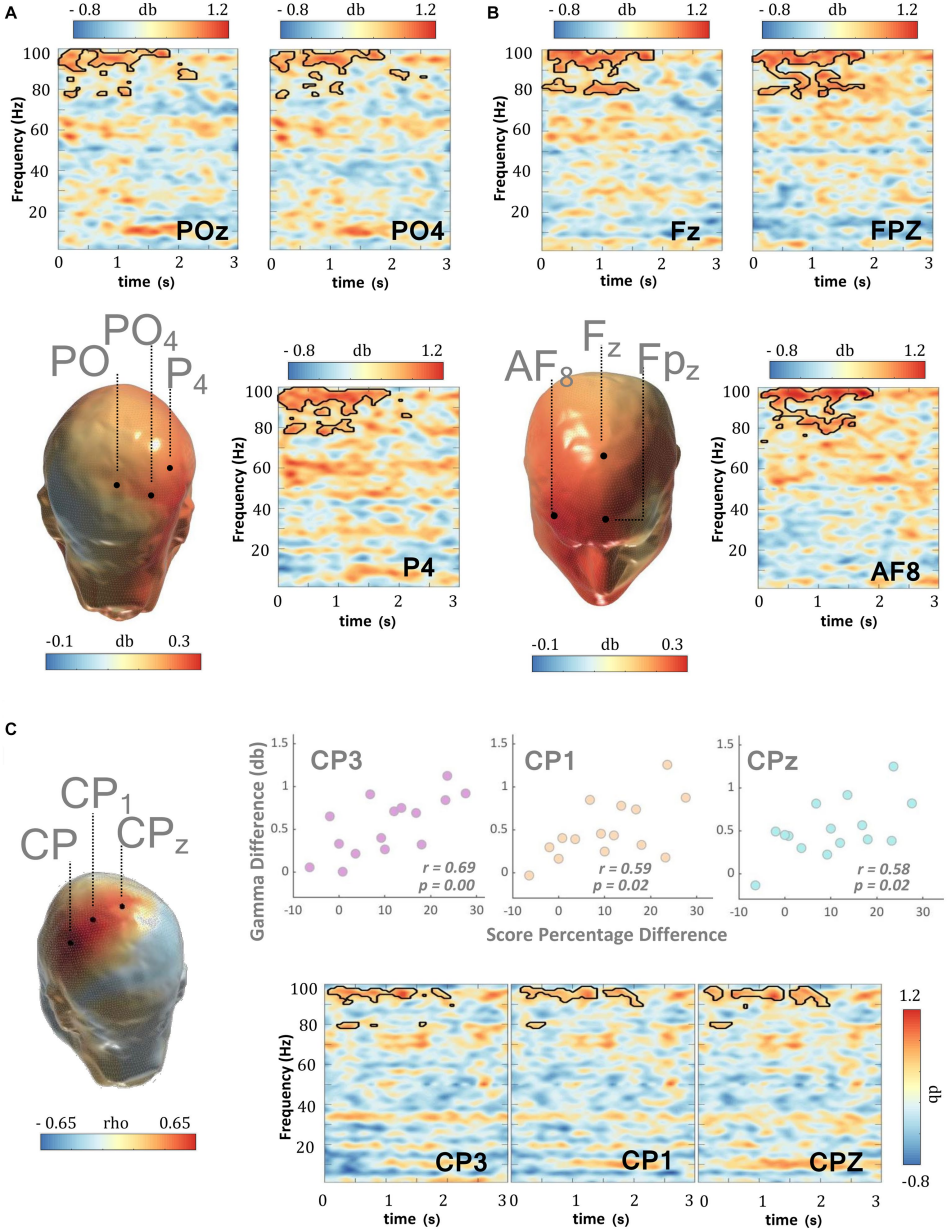
WM delay gamma and MPH. When the delay period (0–3,000 ms) activity of the DMTS task was compared across medication conditions, MPH triggered significant gamma enhancements within the **(A)** parietal and **(B)** frontal scalp regions of subjects with ADHD. **(C)** The MPH-induced delay gamma enhancements in the left central parietal scalp regions were significantly and positively correlated with the corresponding changes in DMTS score. Scalp maps reflect the averaged gamma power (30–100 Hz) during the 0–3,000 ms window following delay period onset. The outline in the spectrograms demarcates gamma frequencies where statistically significant (two-sided test*, p* < 0.025) enhancements were recorded.

MPH is the frontline treatment of ADHD, ameliorating the ADHD-related symptomatic profile and a number of behavioral deficits, which include WM performance deficits as recorded herein. As such, in the next analysis, we investigated whether MPH might be able to reverse the gamma amplitude discrepancies that were observed when TD subjects were compared with patients. Findings revealed that the gamma amplitude in TD subjects was not statistically stronger than that recorded in patients in the ‘ON-medication’ condition (cluster statistic = 1056.8, corrected *p* = 0.572, SD = 0.011, Cohen’s U1 = 0.1) (Figures 3C,D, Supplementary Figure S3). Indeed, MPH medication appears to trigger a direct and significant enhancement and thus normalization in delay-related gamma activity and this was also confirmed when the same patients were compared across medication conditions (cluster statistic = 15,901, corrected *p* = 0.024, SD = 0.003, Cohen’s U1 = 0.5) (Figures 4A,B, Supplementary Figure S4). Studies have implied a positive association between MPH and general arousal effects (Looby and Earleywine, 2011) with the latter substantiated by an increase in gamma power (Balconi and Lucchiari, 2008). If the gamma effect revealed herein relates with general arousal, then it is expected that such might be particularly evident during the prestimulus period when subjects are not directly engaged in a specific cognitive function. When the prestimulus period of both MPH medication conditions were compared, no significant increments (cluster statistic = 9.152, corrected *p* = 0.455, SD = 0.011, Cohen’s U1 = 0.1) or decrements (cluster statistic = −2595.0, corrected *p* = 0.101, SD = 0.007, Cohen’s U1 = 0.1) emerged across the gamma band.

To examine the link between MPH-induced gamma enhancement and WM performance in more detail, we averaged the raw power differences across medication conditions in the gamma frequencies where statistical differences across conditions were observed (inside the statistical masks), and correlated them with the corresponding MPH-induced changes in the DMTS task percentage score. Figure 4C highlights the significant and positive correlation between the changes in DMTS task score and the corresponding changes in gamma amplitude across medication conditions. This association was recorded in the central-parietal electrodes: CP3 (*r* = 0.69, *p* = 0.00), CP1 (*r* = 0.59, *p* = 0.02) and Cpz (*r* = 0.58, *p* = 0.02), further implying that the gamma enhancement triggered by MPH is statistically associated with the concomitant improvement in DMTS task percentage score.

#### 3.2.4. PAC explains instances of delay-related gamma activity

**Figure 5:**
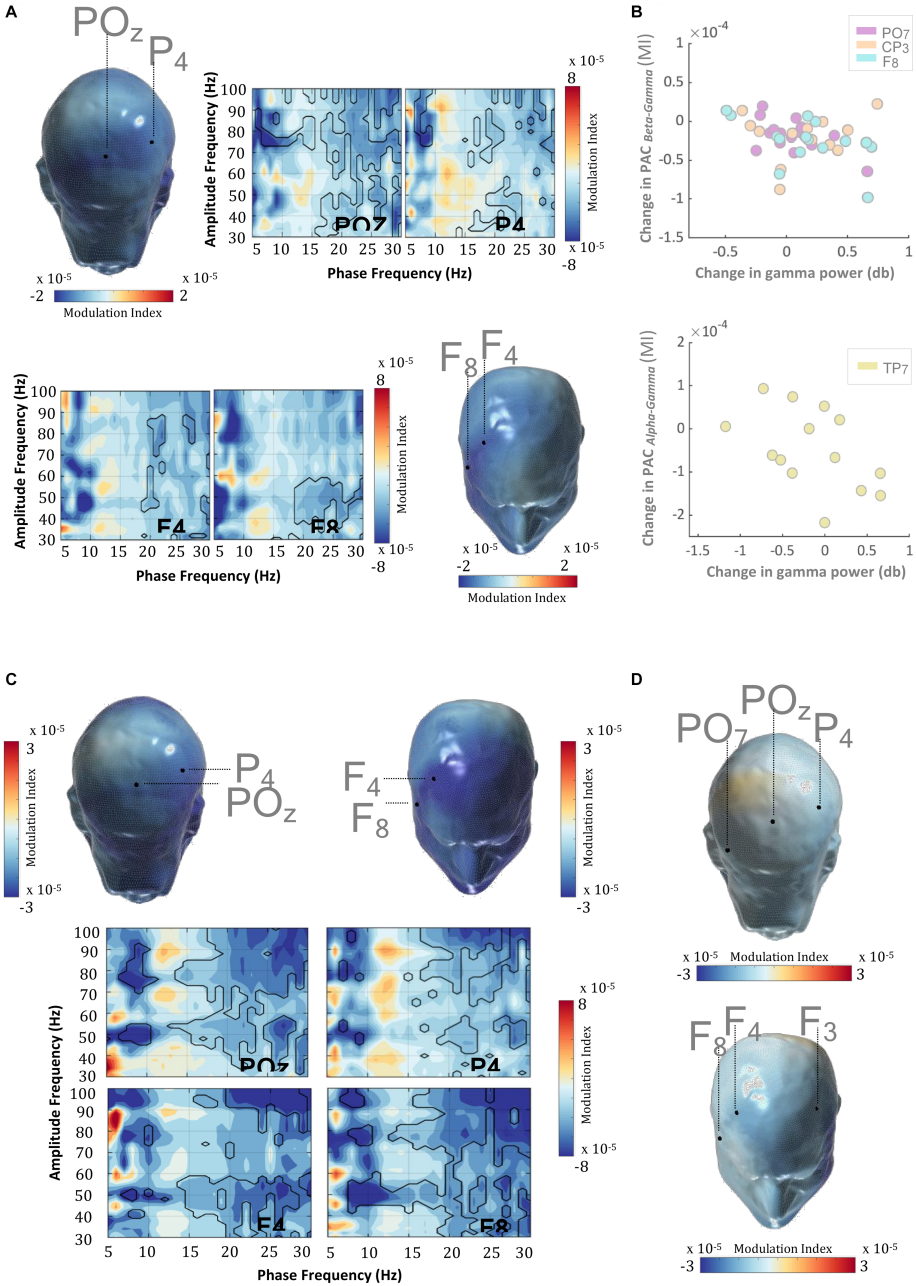
WM delay PAC and MPH. **(A)** In ADHD subjects, MPH induced a direct and significant decrease in frontal-parietal PAC during the delay period (0–3,000 ms) of the DMTS task. This PAC decrement emerged between the phase of the alpha/beta rhythm and amplitude of the gamma rhythm. Scalp PAC profiles display the average modulation index (MI) across statistically significant phase (7–20 Hz) and amplitude frequencies (30–100 Hz). **(B)** MPH-induced decrements in delay alpha-gamma and beta-gamma PAC were significantly and negatively correlated with the MPH-induced gamma amplitude enhancements. This effect was recorded in electrodes: PO7, CP3, F8, and TP7. **(C)** The coupling between the beta phase and gamma amplitude in TD was decreased when compared with ADHD subjects in the “OFF” medication condition. Scalp PAC profiles display the average modulation index (MI) across statistically significant phase (7–20 Hz) and amplitude frequencies (30–100 Hz). **(D)** There were no significant differences in the frontal-parietal PAC coupling between TD and ADHD subjects when under MPH. Scalp topographies showing MPH-induced normalization of PAC profiles across TD and ADHD subjects. Scalp PAC profiles display the average modulation index (MI) across alpha/beta phase frequencies (7–20 Hz) and gamma amplitude frequencies (30–100 Hz). The outline in the comodulograms demarcates statistically significant (two-sided test*, p* < 0.025) PAC decrements.

Phase amplitude coupling (PAC) (Tort et al., 2010) quantifies the relationship between the phase of slower frequency signals and the amplitude of higher frequency signals. Recent studies have suggested various physiological mechanisms that could possibly explain the amplitude modulation in the higher frequency oscillations by the phase of the slower ones. Among such, PAC between gamma and alpha/beta rhythms has been suggested to play a role in the regulated gating/un-gating of memory representation during WM retention (Bastos et al., 2018). In light of this and other studies that posit WM-related alpha/beta anomalies in ADHD (Zammit and Muscat, 2019), we set out to test whether the MPH-induced gamma power enhancement evidenced herein could be partially explained by the concomitant changes in the alpha/beta-gamma PAC profile. Figure 5A and Supplementary Figure S7 show that MPH evoked a significant reduction in PAC during WM-retention, suggesting that the alpha/beta phase-induced modulation (among others) of gamma amplitude is reduced when patients are under MPH, relative to when not (cluster statistic = −7,965, corrected *p* = 0.024, SD = 0.003, Cohen’s U1 = 0.5).

This result raises the question of whether the MPH-induced PAC modulation is a normalizing effect and so in the subsequent analyses, we contrasted the PAC profiles of the TD group with those of ADHD patients during the “ON” and “OFF” medication conditions. Results in Figures 5C,D and Supplementary Figures S5, S6 revealed a significantly weaker alpha/beta-gamma PAC (among others) in TD, relative to ADHD in the ‘OFF’ medication condition (cluster statistic = −18,793, corrected *p* = 0.010, SD = 0.002, Cohen’s U1 = 0.4), which then was reversed when the same patients were in the “ON” medication condition (cluster statistic = −1735.4, corrected *p* = 0.106, SD = 0.007, Cohen’s U1 = 0.1). The normalization trend appears to share an overlap with the one obtained when retention-induced gamma amplitude perturbations were analyzed, even though these were reversed in the opposite direction.

Alpha-beta rhythms have been suggestive of a reflection of a pervasive inhibitory influence and as such, the reduction of alpha/beta-gamma PAC might appear concordant with the concomitant MPH-induced enhancement of gamma power. This, however, does not provide any direct evidence on the extent to which the observed reduction in PAC might relate to the MPH-induced gamma enhancement as outlined above. The design of the study allowed us to address this question by correlating MPH-induced changes in alpha/beta-gamma PAC with the MPH-induced changes in gamma amplitude within the same subjects. We conducted a separate analysis for the alpha (7–14 Hz) and beta (15–20 Hz) frequency bands, sampling the amplitude of those specific gamma frequencies that showed significant coupling differences with the alpha or beta frequencies. Results revealed a significant and negative correlation for both beta and alpha frequency bands. Indeed, the MPH-induced decrease in beta-gamma and alpha-gamma PAC was significantly and inversely related with the corresponding gamma amplitude enhancement observed across medication conditions (Figure 5B, Table 4). This finding was evident in both parietal and frontal scalp regions and suggests a mechanistic correlate to support delay-related gamma activity and its catecholaminergic modulation during WM.

**Table 4:** Significant correlations between MPH-induced changes in alpha-gamma PAC/beta-gamma PAC and MPH-induced changes in the amplitude of PAC-modulated gamma frequencies.

Electrode	Frequency band	Correlation coefficient (*r*)	*p*-value
TP7	Alpha (7–14 Hz)	−0.576	0.024
PO7	Beta (15–20 Hz)	−0.573	0.026
CP3	Beta (15–20 Hz)	−0.533	0.040
F8	Beta (15–20 Hz)	−0.530	0.042

## 4. Discussion

Neural oscillations are partitioned into various bands, laminar profiles and above all cognitive functionality. There is much interest to understand how these different bands function, interact and in turn, regulated. Here, we have focused on WM delay gamma to investigate its coupling with the alpha/beta rhythms and above all its neuromodulation by MPH. We addressed this through the use of both human control and ADHD patient subjects, which revealed novel electrophysiological deficits in ADHD, as well as novel MPH-induced normalization of both gamma amplitude and its coupling with the alpha/beta rhythms. Decreased alpha/beta-gamma coupling is known to facilitate memory representations by withdrawing the default suppression of gamma ensembles coding the maintained stimuli (Lundqvist et al., 2016; Bastos et al., 2018). Here, we present EEG evidence which suggests that these dynamics are sensitive to catecholaminergic neuromodulation. MPH decreased alpha/beta-gamma coupling and this was related to the increase in delay-relevant gamma activity evoked by the same drug. These results add further to the neurophysiological findings that reflect an electrophysiological dimension to the well-known link between WM delay and catecholaminergic transmission.

The ability to represent and manipulate information that has dissipated from the sensorium is crucial for most, if not all, sophisticated cognition. Many efforts have been made to characterize the neural dynamics that support memory retention, and this has been most often done by employing WM delay tasks, such as the DMTS task (Sreenivasan and D’Esposito, 2019). DMTS tasks induce a temporal segregation between the delay and other WM stages, thus facilitating the isolation of delay-related neural activities with more accuracy. Laminar recordings conducted in primates during the DMTS task revealed that delay-related activity is characterized by its superficial origin and gamma rhythmicity (Bastos et al., 2018). Of particular interest is the finding that implicates the persistence of sensory-evoked gamma activities into the delay period of the DMTS task, when the sensory stimulus is withdrawn (Bastos et al., 2018). In this same study, the superficial gamma activity recorded during the delay period was found to encode stimulus information, which thus implied an overlap between sensory-evoked and delay gamma activities. Human studies employing high-field fMRI overlap with these studies in that the delay period activity is similarly sampled from superficial layers (Finn et al., 2019). EEG electrodes record volume conducted oscillating activity originating from multiple cortical laminae (Scheeringa and Fries, 2019). The overlapping superficial sources for both delay activity and human EEG gamma activity (Scheeringa et al., 2016) appears to suggest that some aspect of the human gamma activity might be perturbed during the delay period of the DMTS task.

We teased out this possibility by conducting human EEG recordings during DMTS and WM-CTR tasks. The induced gamma activity recorded in this study was shown to be the only spectral signature within the 1–100 Hz bandwidth analyzed that distinguished DMTS from WM-CTR delay activity. This finding corroborates those of previous studies (Tallon-Baudry et al., 1998; Khursheed et al., 2011; Roux et al., 2012) and suggests that WM maintenance triggers selective modulation in gamma rhythmicity. The comparison with the WM-CTR task also supports the notion that the induced gamma effect was a unique memory effect and not the product of circuit/network effects that follow stimulus cessation (Traub et al., 2020). Although the scalp maps appear to reveal stronger gamma rhythmicity in the right-frontal and parietal electrodes, however this difference was not statistically significant, which is in contrast to previous research demonstrating right hemispheric effects for visual memory (Wang and Ku, 2018; Hilbert et al., 2019).

The use of clinical patients assumed to be associated with specific neurotransmission deficits provides a limited, yet non-invasive window to investigate the neuromodulation of human oscillatory activity. This might be particularly relevant when such patients are administered psychotherapeutic drugs that attempt to correct the same neurotransmission deficits associated with the pathology. ADHD patients have been predominantly characterized by a catecholaminergic dysfunction, as in fact, much of the associated symptomology dissipates following the administration of the catecholamine transporter blocker MPH (Prince, 2008). MPH has a short half-life, enough to permit EEG recordings conducted “under” and “not-under” medication effects within the same period and above all within the same patient. Catecholamines are known to exert profound modulatory effects on WM (Arnsten, 2011). Studies have indeed shown that such effects, mediated by both dopaminergic and noradrenergic neuromodulation (Berridge and Stalnaker, 2002) might be particularly relevant to delay-related activity (Zhang et al., 2013; Wang et al., 2019). Both neurotransmitters enhance delay activity with a positive effect on WM performance (Clark and Noudoost, 2014). The latter is also likely to suggest that MPH effects on WM might in part depend upon baseline catecholamine neurotransmitter concentrations. In fact, earlier studies have presented neurophysiological evidence that supports a positive relationship between MPH effects and baseline catecholamine levels (Volkow et al., 2002). While this relationship has also been recorded for cognitive ability levels, in particular, a synergistic relationship between WM capacity and MPH-induced reward learning (van der Schaaf et al., 2013), it further implies that MPH effects are not limited to clinical cohorts, such as ADHD, as indeed MPH-induced cognitive ‘enhancements’ extend to healthy subject populations, as has already been demonstrated (Cools et al., 2009).

In this study, we address the link between MPH-induced catecholaminergic activity and WM by raising the question of whether this could be, in part, explained by a corresponding modulation of delay-gamma activity. Herein, when the ADHD patients were compared during the “ON” and “OFF” MPH conditions, MPH evoked a significant enhancement in the delay-related gamma activity, which in several instances, was statistically associated with better DMTS performance. In fact, MPH triggered a significant normalization of both delay-gamma deficits and DMTS performance when the same patients in the ‘OFF’ MPH condition were compared with age-matched controls. The latter result in a way elaborates previous studies that established a link between MPH effects and WM capacity (van der Schaaf et al., 2013), by suggesting a non-linear interaction between WM capacity and MPH effects, as indeed, the significant MPH effects herein were recorded from patients with significantly weaker WM capacity relative to age-matched controls.

ADHD patients have been associated with various oscillatory deficits recorded during both resting and event-related states (Lenartowicz et al., 2018), however, and to our knowledge this is the first study that supports a relationship between ADHD and WM delay-related gamma deficits. This latter interpretation should however be pursued with a degree of caution as gamma decrements might in effect constitute “medication wear-off effects,” in particular, stimulant rebound withdrawal effects (worsening of symptoms beyond baseline, e.g., see Cox et al., 2004; Lerner and Klein, 2019). That said, while there is a lack of studies investigating MPH rebound in ADHD, to our knowledge, these have not been conducted during specific WM scenarios (Lerner and Klein, 2019). The fact that MPH rebound in ADHD is substantiated by ADHD symptom recurrence (Cox et al., 2004; Lerner and Klein, 2019) implies that the gamma anomalies recorded herein bear a relationship with ADHD pathology even if such might in effect entail MPH withdrawal effects. The increase in gamma power following MPH administration suggests that the changes in the gamma activity, recorded in the ADHD cohort herein are more likely than not related to the catecholaminergic dysregulation associated with the pathology. The gamma deficits in the ADHD patients herein appeared across both left and right electrode clusters at statistically similar levels, thus providing no evidence in favor of the right hemispheric dysfunction associated with ADHD (Stefanatos and Wasserstein, 2001). However, such interpretations should be pursued with additional caution, as on one side, gamma activity cannot be directly equated with imaging activity and secondly, the lateralized deficits theory in ADHD is more likely a reflection of the impaired cognitive processes that impose their own footprints on brain activation patterns (Hale et al., 2009). Thirdly, as recently suggested, rather than a lateralized deficit, a dorsal to ventral imbalance along the superior longitudinal fasciculus might better account for the behavioral and oscillatory deficits in ADHD (Mazzetti et al., 2022).

Studies have put forth the hypothesis that alpha/beta to gamma coupling might explain much of the gamma perturbations observed during WM delay (Lundqvist et al., 2016; Bastos et al., 2018). In this scenario, alpha/beta oscillations are reflective of the inhibitory perturbations of the underlying circuitry in which a gating mechanism regulates access to the superficial cortical layers. In turn, a decrease in alpha/beta to gamma coupling during WM delay (Bastos et al., 2018) is hypothesized to release the inhibitory influences upon superficial layers, strengthening and thus allowing gamma cycles to code and maintain sensory information. If MPH in our study triggered a selective and significant delay gamma enhancement, it is then reasonable to suggest that this should also be supplemented by a corresponding decrease in alpha-beta coupling. Our findings do concur with this possibility as MPH triggered a concomitant and significant decrease in alpha/beta to gamma coupling. As was the case with gamma amplitude deficits, the MPH-induced PAC modulation was one of a normalization effect. In fact, MPH decreased alpha/beta-gamma PAC in patients to the level that was not statistically different from that recorded in controls. Although the normalization of both gamma amplitude and PAC would suggest some form of correlation, however, this does not specify the extent to which the MPH-induced gamma enhancement is related to the observed PAC reduction. Here, we demonstrate that the MPH-induced changes across gamma amplitude and PAC were significantly and inversely related, implying cross frequency coupling in the MPH-induced gamma modulation.

It thus appears that alpha/beta rhythms inhibit gamma rhythmicity with important consequences to stimulus coding and WM maintenance. Whether this coupling was interlaminar or not, as is the case in primates, remains to be seen, however, it appears that the results herein would seem to support this notion with the addition that such a mechanism might be sensitive to catecholaminergic neuromodulation. There have been various interesting attempts to incorporate these electrophysiological observations with already existing interlaminar WM models (reviewed in Miller et al., 2018). The data obtained herein does not permit one to speculate on the circuit mechanisms that relate to gamma modulation, alpha/beta-gamma coupling and catecholamines. However, some tentative predictions might be explicated and tested in prospective studies. Decades of pharmacological research have contributed to a wealth of information regarding the MPH-induced inverted U-dose relationship with WM processing (Spencer and Berridge, 2019) and thus for example in the first instant, how does this relate to the MPH-induced changes in alpha/beta-gamma coupling found herein? Studies suggest that the inverted U-dose response curve is at large the combinatorial effect of various receptors, which most prominently include, the dopamine D1 and adrenergic α2-receptors (Arnsten and Dudley, 2005). It might be of interest then to use this pharmacological context, in addition to the various catecholamine receptor blockers, to elucidate the selective contribution of catecholamine receptors on both delay-gamma amplitude and its coupling with alpha/beta rhythms. This might provide for more fine detail into the various interlaminar circuit architectures proposed (Miller et al., 2018) and above all, an understanding of how specific neurotransmission systems might calibrate the balance between feedforward gamma and feedback alpha/beta processing. Clinically, the coupling between feedforward gamma and feedback alpha/beta might be exploited to refine clinical behavioral descriptions of neuropathology. Framing neuropathology along bottom-up and top-down-related deficits appears to be an appealing foundation to add to the much exhausted “roots of mental illness” (Marshall, 2020). The evolving neurophysiological and neuroanatomical data supplementing such a framework might provide for a tighter overlap between the clinical and neuroscientific dimensions of neuropsychiatric disease and thus that of what one merits of translational medicine.

## Data availability statement

The raw data supporting the conclusions of this article will be made available by the authors, without undue reservation.

## Ethics statement

The studies involving humans were approved by University Research Ethics Committee (UREC), University of Malta. The studies were conducted in accordance with the local legislation and institutional requirements. Written informed consent for participation in this study was provided by the participants’ legal guardians/next of kin.

## Author contributions

NZ: Investigation, Software, Writing – original draft, Data curation, Formal analysis, Visualization. RM: Conceptualization, Funding acquisition, Investigation, Methodology, Project administration, Resources, Supervision, Validation, Writing – review & editing, Writing – original draft.

## Abbreviations

WM: Working memory
DMTS: Delayed-matching to sample task
ADHD: Attention deficit hyperactivity disorder
EEG: Electroencephalogram
TD: Typically developing
MPH: Methylphenidate
ICA: Independent component analysis
IC: Independent components
PAC: Phase amplitude coupling
