# Supplementary material for: Alpha/beta-gamma decoupling in methylphenidate medicated ADHD patients
Source: Front Neurosci. 2023 Sep 29;17:1267901. doi: 10.3389/fnins.2023.1267901 (PMC10570420; doi:10.3389/fnins.2023.1267901)
Supplement: Supplementary file 1 [file Data_Sheet_1.PDF]

Table 1: Sample demographics and symptoms rated by Conners parent and teacher rating scales.

SUBJECT TYPE	N	AGE	T_{OPP}	T_{ATT}	T_{HYP}	T_{GLO}	P_{OPP}	P_{ATT}	P_{HYP}	P_{GLO}
TD	15	13.13	55.7	51.1	55.7	51.9	56.8	53.9	56.5	50.1
ADHD	15	13.39	76.1	69.2	80.0	75.0	75.4	69.7	82.7	73.7

$\mathbf{T}_{\boldsymbol{OPP}}$: Conners teacher oppositional index; $\mathbf{T}_{\boldsymbol{ATT}}$: Conners teacher attentional index; $\mathbf{T}_{\boldsymbol{HYP}}$: Conners teacher hyperactivity index; $\mathbf{T}_{\boldsymbol{GLO}}$: Conners teacher global index; $\mathbf{P}_{\boldsymbol{OPP}}$: Conners parent oppositional index; $\mathbf{P}_{\boldsymbol{ATT}}$: Conners parent attentional index; $\mathbf{P}_{\boldsymbol{HYP}}$: Conners parent hyperactivity index; $\mathbf{P}_{\boldsymbol{GLO}}$: Conners parent global index.

Table 2: Laterality effects for the gamma band (30-100 Hz) between the DMTS and WM-CTR task.

P-value		Cluster statistic		SD		Cohen's U1	
CI_{Pos}	CI_{Neg}	CI_{Pos}	CI_{Neg}	CI_{Pos}	CI_{Neg}	CI_{Pos}	CI_{Neg}
0.03	0.94	1117.4	-161.4	0.004	0.005	0.07	0.07

Left-sided electrodes (*n=13*): PO7, PO3, P7, P3, P1, TP7, CP3, CP1, FT7, FC3, F7, F3, AF7; **Right-sided electrodes** (*n=13*): PO8, PO4, P8, P4, P2, TP8, CP4, CP2, FT8, FC4, F8, F4, AF8. $\mathbf{CI_{Pos}}$: Largest positive cluster; $\mathbf{CI_{Neg}}$: Largest negative cluster. The analysis shows that the comparison was not statistically significant (two-sided test, p >0.025).

Table 3: Laterality effects for the gamma band (30-100 Hz) between the TD and ADHD subjects in the 'OFF-medication condition.

P-value		Cluster statistic		SD		Cohen's U1	
CI_{Pos}	CI_{Neg}	CI_{Pos}	CI_{Neg}	CI_{Pos}	CI_{Neg}	CI_{Pos}	CI_{Neg}
0.93	0.99	69.1	-22.96	0.005	0.002	0.03	0.03

Left-sided electrodes (*n=13*): PO7, PO3, P7, P3, P1, TP7, CP3, CP1, FT7, FC3, F7, F3, AF7; **Right-sided electrodes** (*n=13*): PO8, PO4, P8, P4, P2, TP8, CP4, CP2, FT8, FC4, F8, F4, AF8. $\mathbf{CI_{Pos}}$: Largest positive cluster; $\mathbf{CI_{Neg}}$: Largest negative cluster. The analysis shows that the comparison was not statistically significant (two-sided test, p >0.025).

Table 4: Significant **c**orrelations between MPH-induced changes in alpha-gamma PAC/ beta-gamma PAC and MPH-induced changes in the amplitude of PAC-modulated gamma frequencies.

	Electrode	Frequency band	Correlation coefficient (*r*)	p-value
	TP7	Alpha (7-14 Hz)	-0.576	0.024
	PO7	Beta (15-20 Hz)	-0.573	0.026
	CP3	Beta (15-20 Hz)	-0.533	0.040
	F8	Beta (15-20 Hz)	-0.530	0.042
